# Precision Channel Engineering of Nanotube-Embedded Organic Electrochemical Transistors for Ultrasensitive Neurofilament Light Chain Detection

**DOI:** 10.1021/acsabm.5c02404

**Published:** 2026-01-23

**Authors:** Jia-Wei She, Lu-An Lin, Jayakrishnan Aerathupalathu Janardhanan, I-Chen Wang, Feng-Chen Hsu, Hsueh-Sheng Tseng, Yu-Sheng Hsiao, Hsiao-hua Yu

**Affiliations:** † Smart Organic Materials Laboratory, Institute of Chemistry, 38017 Academia Sinica, No. 128, Section 2, Academia Road, Nankang, Taipei 11529, Taiwan; ‡ Taiwan International Graduate Program (TIGP), Nano Science & Technology Program, Academia Sinica, No. 128, Section 2, Academia Road, Nankang, Taipei 11529, Taiwan; § Department of Engineering and System Science, National Tsing Hua University, No. 101, Section 2, Guangfu Road, East District, Hsinchu City 300, Taiwan; ∥ College of Medicine, 56081 Chang Gung University, No. 259, Wenhua First Rd., Guishan Dist., Taoyuan City 33302, Taiwan; ⊥ Department of Materials Science and Engineering, 34878 National Taiwan University of Science and Technology, No. 43, Sec. 4, Keelung Rd., Taipei 106335, Taiwan

**Keywords:** organic electrochemical transistor, poly(3,4-ethylenedioxythiophene), bioelectronics, neurofilament light chain, nanostructure, biosensors

## Abstract

The quantitative monitoring of neurofilament light chain (Nf-L) is critical for the early diagnosis and prognosis of neurodegenerative disorders, such as amyotrophic lateral sclerosis (ALS), yet achieving femtomolar sensitivity in a portable, label-free format remains a formidable challenge. Here, we report a high-performance organic electrochemical transistor (OECT) immunosensor engineered via the precise template-free electropolymerization of a dual-functional poly­(EDOT–COOH–*co*-EDOT-EG3) copolymer. By systematically modulating the polymerization kinetics, we elucidated a decisive structure–function relationship governing biosensing efficacy: while microstructured channels formed at longer deposition times exhibited superior intrinsic transconductance due to maximized volumetric capacitance, the optimized nanotubular architecture provided the ideal balance of open porosity and accessible surface area. This specific nanotopography facilitated a significantly higher density of covalent antibody immobilization compared to its microstructured counterpart, thereby dominating the signal transduction mechanism through enhanced dielectric barrier formation upon antigen binding. Capitalizing on this morphology-governed sensitivity, the platform achieved a theoretical limit of detection (LOD) of 0.062 fg/mL (3σ criterion) and a rigorous LOD of 32.77 fg/mL (Hubaux-Vos method) across a broad dynamic range, along with exceptional selectivity and operational stability over 500 cycles. These findings underscore the critical role of precision channel engineering in bioelectronics, establishing a robust, lithography-free pathway for next-generation point-of-care diagnostics targeting diseases.

## Graphical abstract

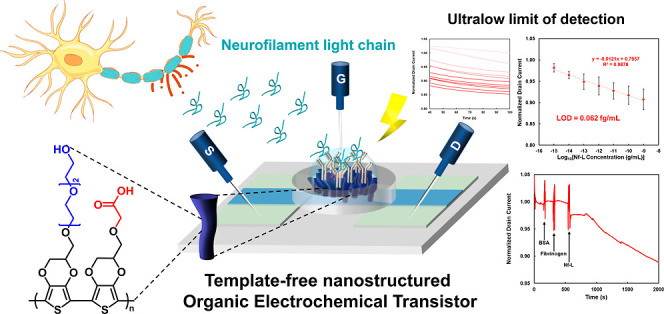

## Introduction

In recent years, many studies have suggested that neurofilament light chain (Nf-L), the cytoskeleton protein, is a promising diagnostic, prognostic, and monitoring biomarker for neurodegeneration, inflammation, and neuroaxonal damage,^1–4^ including amyotrophic lateral sclerosis (ALS), multiple sclerosis (MS), and Parkinson’s disease (PD).^5^ Neurofilaments (NFs) are heteropolymers and type IV intermediate filaments^6^ composed of four subunits: neurofilament heavy chain (NF–H, *M*_w_ = ∼200 kDa), neurofilament medium chain (NF-M, *M*_w_ = ∼150 kDa), Nf-L (*M*_w_ = ∼68 kDa), and α-internexin or peripherin.^4,6,7^ Despite differences in molecular weight and function, all neurofilament (Nf) proteins share a conserved structure comprising a central α-helical rod domain, a variable N-terminal domain, and a C-terminal tail that varies in length. Notably, Nf-L is the most abundant and soluble form of neurofilaments,^6,8^ and mutations promoting its aggregation are associated with motor neuron impairment.^9^ Moreover, when neurons or axons are damaged, Nf-L is released into the extracellular space and eventually into cerebrospinal fluid (CSF) and blood. Thus, elevated Nf-L levels in CSF and blood can be detected and therefore serve as a general robust biomarker for detecting neurological disorders.^5,10^

The utility of Nf-L becomes especially evident in neurodegenerative diseases such as ALS, where early and accurate detection remains a critical unmet need. This fatal neurodegenerative disorder is characterized by progressive degeneration of upper and lower motor neurons, resulting in muscle weakness, atrophy, respiratory failure, and a median survival of only 3–5 years after symptom onset.^11^ Early ALS symptoms often overlap with other neuromuscular conditions, thereby causing misdiagnosis and substantial diagnostic delays.^12^ Currently, diagnosis of ALS remains a “diagnosis of exclusion” that requires 10–15 months from initial symptom presentation to confirmation. Diagnosing ALS through genetic testing remains challenging due to its largely sporadic nature, genetic heterogeneity, and interactions with environmental factors. Since many mutations are nonspecific or asymptomatic, their diagnostic value is limited.^10^ The lack of robust biomarkers and analytical tools further impedes timely diagnosis and interventions.^13^ Consequently, significant efforts have directed toward identifying reliable biomarkers in various bodily fluids, including CSF, plasma, serum, and blood, to aid in ALS diagnosis, prognosis, and disease monitoring. Among these, Nf-L has shown particular promise as a robust diagnostic and prognostic biomarker.^3,14–16^ In CSF, it demonstrates high accuracy for distinguishing ALS from controls and correlates strongly with survival outcomes. In plasma, Nf-L levels remain stable throughout disease progression and are significantly elevated in ALS patients compared to healthy controls and mimics. Furthermore, early stage elevation effectively predicts aggressive disease progression and poorer clinical outcomes.

Conventional ELISA methods have demonstrated high sensitivity for detecting Nf-L in CSF. However, since Nf-L concentrations are substantially lower in blood, their use have been limited to CSF samples obtained via invasive lumbar puncture.^17^ Other widely adopted immunoassays include single molecule enzyme-linked immunosorbent assays (SiMoA),^18,19^ Lumipulse, and Ella,^20^ all of which have been extensively tested in clinical samples across various neurological disorders and have shown high sensitivity for Nf-L detection.

Despite significant advances, conventional immunoassays remain constrained by high costs, the reliance on secondary antibody labeling, and limited portability. These challenges have catalyzed interest in electrochemical assays, which offer rapid, label-free detection with minimal setup, which are key prerequisites for point-of-care diagnostics. Among these platforms, organic electrochemical transistors (OECTs) based on poly­(3,4-ethylenedioxythiophene):polystyrenesulfonate (PEDOT:PSS) have emerged as a premier class of bioelectronic interfaces (BEIs), owing to their intrinsic signal amplification,^21^ ease of miniaturization,^22^ and biocompatibility. However, achieving ultralow detection limits and long-term reliability requires precise modulation of the polymer interface. Our group has demonstrated that tailoring the side-chain functionality and nanotopology of PEDOT significantly enhances sensor performance. For instance, we engineered nanotubular poly­(EDOT–COOH–*co*-EDOT-EG3) architectures to create a sweat cortisol sensor with an ultralow LOD of 0.0088 fg/mL and a shelf life exceeding 20 days.^23^ Similarly, we utilized this functionalized nanotube strategy to develop flexible nanoelectrode platforms for Neuropeptide-Y detection, achieving an LOD of 0.68 pg/mL with high selectivity against interfering cytokines.^24^ Most recently, we advanced this approach by fabricating PEDOT nanorod arrays via oxidative trans-printing; this 3D-OECT platform enabled the rapid identification of SARS-CoV-2 spike proteins in artificial saliva with an LOD of 138 fM, demonstrating exceptional stability and accuracy in complex biological matrices.^25^

Beyond infectious diseases and stress markers, OECTs have been extensively explored for the quantitative monitoring of neurodegenerative disease biomarkers, particularly amyloid-β (Aβ) aggregates and dopamine.^26–28^ Notably, Koklu et al. engineered a microfluidic-integrated OECT platform capable of detecting Aβ aggregates in human serum with a LOD as low as 100 zM.^29^ While dopamine sensing remains a prominent area of investigation,^26^ recent attention has shifted toward Nf-L, a critical indicator of neuronal damage. In this domain, related transistor architectures have demonstrated promising results. For instance, Lin et al. developed an extended-gate field-effect transistor (EGFET) to quantify neuron-derived exosomes in plasma from Alzheimer’s patients, achieving an LOD of 60 exosomes/mL.^30^ Furthermore, EGOFETs have been successfully employed for direct Nf-L protein detection, reaching a sensitivity of 30 fM.^9^ Despite these promising results using FET-based platforms, the application of OECTs, with their superior transconductance and biocompatibility, for ultrasensitive Nf-L detection remains underexplored.

Addressing this unmet need, we report a nanotube-engineered OECT platform constructed through the template-free electropolymerization of poly­(EDOT–COOH–*co*-EDOT-EG3) within the active channel. In this system, EDOT-COOH facilitates the covalent tethering of anti-Nf-L antibodies, while EDOT-EG3 effectively minimizes nonspecific fouling. To explicitly elucidate the sensing enhancement driven by nanotopography, a microstructured control device was fabricated and evaluated in parallel. The resulting nanotube-engineered OECT sensor delivers exceptional analytical performance, achieving real-time detection over a broad dynamic range (1 fg/mL to 1 ng/mL) with an ultralow LOD of 0.062 fg/mL (3σ criteria) and a rigorous LOD of 32.77 fg/mL (Hubaux-Vos method). Exhibiting excellent linearity, selectivity, and stability, this platform demonstrates significant potential for clinical translation and integration into next-generation point-of-care diagnostics.

## Experimental Section

### Materials

A comprehensive list of reagents and chemicals is provided in the Supporting Information.

### OECT Device Fabrication and Channel Modification

OECT devices were constructed on indium tin oxide (ITO)–coated glass substrates (2 cm × 2 cm) using a streamlined, lithography-free protocol. The active channel architecture comprised a bilayer structure: a spin-coated PEDOT:PSS underlayer and an electrochemically engineered top layer consisting of either poly­(EDOT–COOH–*co*-EDOT-EG3) nanotubes or microstructured films. To ensure independent control over material properties, each layer was fabricated using distinct deposition techniques. First, the PEDOT:PSS underlayer was deposited onto the channel region via spin-coating at 1000 rpm for 10 s, yielding a uniform thin film. A commercial CO_2_ laser engraving system (Universal VLS 2.30, Universal System, AZ, USA) was subsequently employed to pattern the film, defining precise and well-confined channel boundaries.^24^ Following the formation of this base layer, the functionalized PEDOT copolymer was engineered atop the PEDOT:PSS to serve as the high-surface-area bioelectronic interface.

### Template-Free Electropolymerization of Functionalized Nanostructures

The 3D-nanostructured copolymers were fabricated via in situ electropolymerization using a three-electrode configuration connected to a potentiostat (PGSTAT128N, Autolab). The reaction was confined within a polydimethylsiloxane (PDMS) chamber (volume = ∼50 μL) affixed to the device. The ITO source and drain electrodes served collectively as the working electrode, with an Ag/AgCl electrode and a platinum wire acting as the reference and counter electrodes, respectively. Prior to polymerization, all monomer solutions were rigorously degassed under a nitrogen atmosphere to eliminate oxygen interference. Nanotubular architectures were constructed by dissolving EDOT-COOH (5 mM) and EDOT-EG3 (5 mM) in dichloromethane (CH_2_Cl_2_) containing tetrabutylammonium perchlorate (TBAP, 100 mM) as the supporting electrolyte. A constant potential of +1.2 V (vs Ag/AgCl) was applied at 0–2 °C for 60 s, a condition optimized to induce self-assembled nanotube growth. For comparative control experiments, microstructured copolymer films were synthesized under identical conditions but with an extended polymerization time of 120 s. Following deposition, the modified channels were thoroughly rinsed with acetonitrile (MeCN) and doubly distilled deionized water to remove residual monomers and electrolytes, ensuring a pristine interface for subsequent functionalization.

### Surface Biofunctionalization and Antibody Immobilization

To confer specific biorecognition capabilities, the carboxyl-functionalized nanotube and microstructure channels were activated via carbodiimide coupling chemistry. The devices were incubated in phosphate-buffered saline (PBS, 1×, pH 7.4) containing 0.4 M EDC·HCl (99%) and 0.1 M Sulfo-NHS (98%) for 6 h to convert the surface carboxylic acid groups into reactive NHS-ester intermediates. After rinsing with PBS to remove unreacted reagents, a solution of anti-Nf-L antibody (100 μg/mL) was drop-cast onto the active area and incubated for 3 h, enabling robust covalent tethering of the antibodies to the copolymer scaffold. The immunosensors were finally rinsed with PBS to remove loosely bound proteins, yielding fully functionalized devices ready for sensing.

### Electrical and Electrochemical Characterization

The electrical performance of the OECTs was evaluated using an integrated three-terminal measurement system comprising two source meters (Keysight B1500A and Agilent B2912A) and a switching matrix (Agilent E5250A), controlled via customized LabVIEW software. All measurements were conducted in PBS (1×, pH 7.4) using an Ag/AgCl wire as the gate electrode. To characterize the device transfer properties, the gate voltage (*V*_g_) was swept from −0.8 to +0.8 V at a scan rate of 12.5 mV s^–1^, while maintaining a fixed drain voltage (*V*_d_) of −0.5 V. The transconductance (*g*_m_) was derived by differentiating the drain current (*I*_d_) with respect to the gate voltage (*g*_m_ = ∂*I*_d_/∂*V*_g_) to quantify the ion–electron coupling efficiency and signal amplification capability.

### Real-Time Sensing Protocol for Nf-L Detection

The analytical performance of the OECT immunosensor was assessed via real-time amperometric monitoring of the drain current (*I*_d_ vs time). Prior to sensing, the devices were equilibrated in PBS (1×, pH 7.4) for at least 30 min to establish a stable baseline current. For concentration-dependent measurements, standard Nf-L solutions ranging from 1 fg/m to 1 ng/mL were introduced into the PDMS well. Following a 30 min incubation period, which was selected to ensure sufficient thermodynamic equilibrium for antigen–antibody binding while maintaining a practical assay turnaround time, the device response was recorded under a constant gate voltage (*V*_g_ = 0 V) and drain bias (*V*_d_ = −0.5 V) until the signal reached a steady state. To ensure equilibrium and minimize transient noise, the drain current (*I*_d_) and the baseline current (*I*_blank_) were extracted at *t* = 100 s, where the device current reached a stable steady state. The calibration curve was analyzed within the linear range of 1 fg/mL to 1 ng/mL.

### Repeatability and Stability Assessment

To evaluate the intra-assay repeatability, the device was incubated with either PBS (control) or a representative Nf-L concentration (1 ng/mL). Subsequently, the sensor was subjected to five consecutive measurement cycles. Between each measurement, a standard washing step and a 30 min stabilization period were implemented to ensure thermodynamic equilibrium. The drain current was recorded for each cycle to quantify signal drift and measurement precision. Furthermore, long-term operational stability was assessed by subjecting the device to 500 continuous gating cycles (ON/OFF pulses, *V*_g_ = 0.1 V) in a PBS environment.

## Results and Discussion

### Synthesis of Functionalized Monomers

To construct a precise dual-functional interface, we synthesized tailored monomers to serve as bioconjugation anchors and blocking agents, respectively (Figures S1). First, for antibody immobilization, the carboxylic acid-functionalized monomer (EDOT-COOH) was prepared via a two-step sequence involving the etherification of hydroxymethyl-EDOT (EDOT–OH) with methyl 2-bromoacetate, followed by base-catalyzed hydrolysis to liberate the reactive carboxylic acid moiety. Complementarily, to minimize the nonspecific physical adsorption of proteins, the EDOT-EG3 monomer was synthesized via a precise multistep pathway. Initially, triethylene glycol was monoprotected with a trityl group and subsequently mesylated to generate a reactive intermediate. This intermediate underwent a nucleophilic substitution with EDOT–OH under basic conditions, followed by acidic deprotection using Amberlite IR-120 resin to yield the target hydroxyl-terminated EDOT-EG3. The synthesis of both monomers aligned with our prior reports,^23^ yielding the high-purity monomers essential for the precision electropolymerization step.

### Fabrication of the OECT Base Architecture

To establish a robust and reproducible foundation for the biosensor, we employed a streamlined, lithography-free fabrication protocol that prioritizes interfacial stability while minimizing process complexity (Figure 1). We began by performing a rigorous multistep cleaning process on the ITO substrates to remove surface contaminants and ensure uniformity during subsequent coating and electropolymerization steps. The substrates were ultrasonicated in a detergent solution, rinsed thoroughly with deionized (DI) water, and cleaned with organic solvents, including acetone and isopropanol (IPA). This sequence yielded a pristine, hydrophilic ITO surface favorable for reliable thin-film deposition.

**Figure 1:**
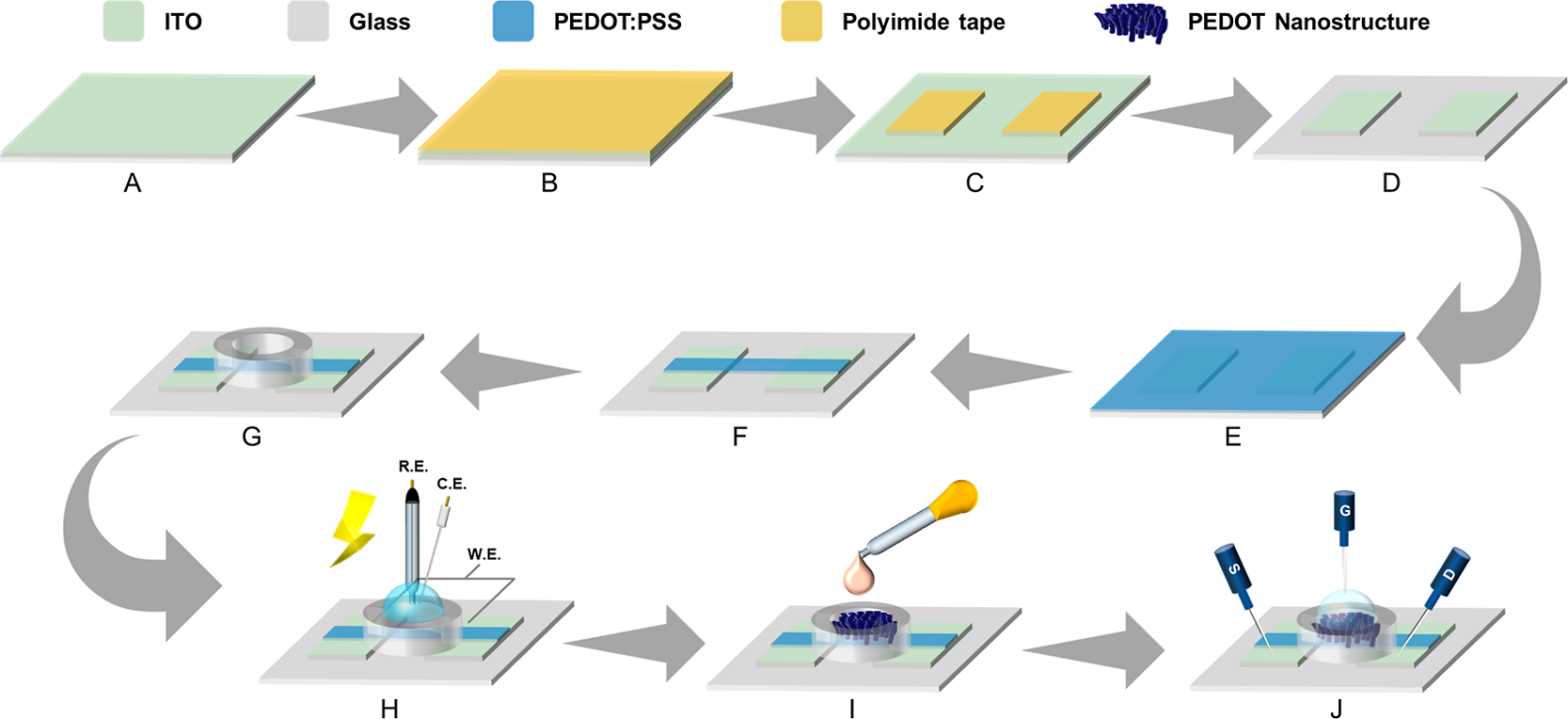
Schematic of the lithography-free fabrication and functionalization workflow. (A–G) Fabrication steps for the OECT base architecture, including electrode patterning and channel isolation. (H) Template-free electropolymerization of PEDOT copolymer nanotubes. (I) Covalent antibody immobilization. (J) Measurement configuration.

To pattern the source and drain electrodes, PI tape was applied as a protective mask, and precise electrode outlines were defined using laser cutting. Crucially, the exposed ITO regions were selectively etched using a controlled mixture of zinc powder and 1 M dilute hydrochloric acid. Compared with conventional high-concentration HCl etching, this milder chemical approach effectively removed the ITO layer while minimizing potential overetching or substrate damage, thereby preserving the structural integrity of the electrode edges.

Following the electrode definition, PEDOT:PSS was spin-coated onto the channel region to establish the initial conductive layer. Spin coating at 1000 rpm for 10 s resulted in a uniform film approximately 201 nm in thickness. To optimize film stability for subsequent aqueous electropolymerization, the devices underwent heat annealing at 140 °C for 1 h in a humid chamber. Finally, a CO_2_ laser was used to ablate excess PEDOT:PSS, yielding a well-defined source–channel–drain configuration, and a PDMS well was affixed to confine the electrolyte. Notably, this optimized workflow obviates the need for expensive, high-maintenance photolithographic instrumentation. By refining the selectivity of the Zn/HCl etching and the thermodynamics of the humidity-assisted annealing, we achieved a substantial improvement in device yield and batch-to-batch reproducibility, providing a reliable platform for precise biosensing.

### Electropolymerization-Controlled Formation of Nano/Microstructures and Morphology-Dependent Electrical Characteristics

While conventional OECT biosensors predominantly focus on functionalizing gate electrodes to confer specificity, our group has pioneered a distinct channel-engineering strategy that directly modifies the conducting polymer layer. Since OECT operation relies on the injection of ions into the polymer volume, a mechanism driven by volumetric capacitance, tailoring the active channel offers a more direct pathway to amplify signal transduction compared to 2D planar gates. We have previously validated the efficacy of this approach in high-performance sensors for sweat cortisol and SARS-CoV-2 spike proteins.^23,25^ Building on these foundations, we engineered a series of morphology-controllable 3D PEDOT copolymer architectures within the active channel to simultaneously maximize the electroactive volume (for intrinsic signal amplification) and the accessible surface area (for antibody anchoring).

In this study, we engineered a series of morphology-controllable three-dimensional (3D) poly­(EDOT–COOH–*co*-EDOT-EG3) copolymer (Figure 2Α) nanostructures within the active channel layer of the OECT to enhance both its electrochemical performance and biosensing capability. This channel-engineering strategy was guided by two central design principles. First, the incorporation of a newly fabricated tubular type bioelectronic interface (BEI) increases the electroactive volume and charge-storage capacity of the channel, thereby improving the intrinsic signal-amplification efficiency of the OECT. Second, manipulating the channel morphology at the nano- and microscale effectively enlarges the accessible surface area, providing a greater density of anchoring sites for anti-Nf-L antibody immobilization and ultimately enhancing antigen–antibody recognition during Nf-L detection.

**Figure 2:**
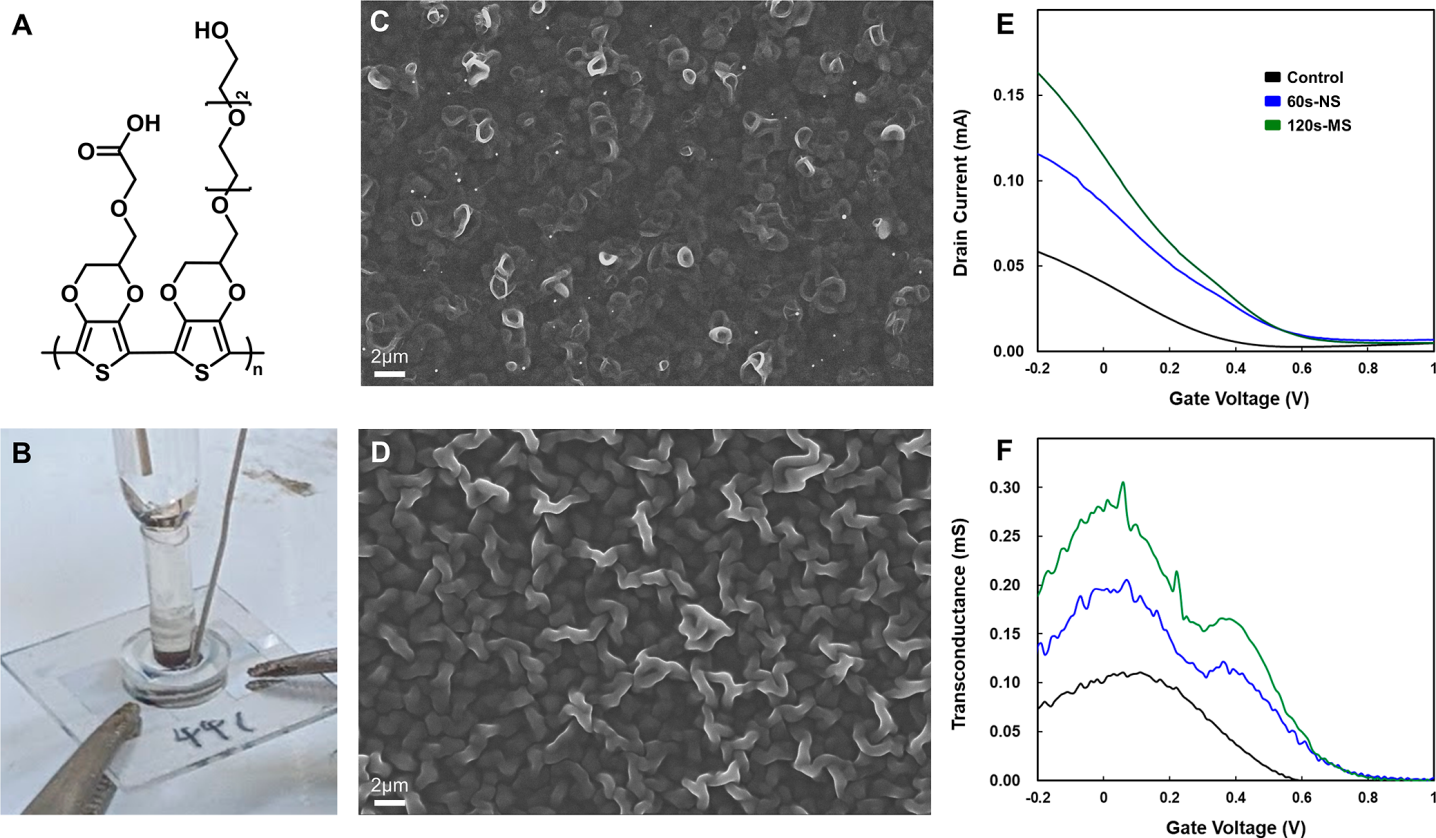
Molecular design, fabrication, and morphology-dependent electrical characteristics of the OECT channel. (A) Chemical structure of the poly­(EDOT–COOH–*co*-EDOT-EG3) copolymer, featuring carboxylic acid sites for bioconjugation and ethylene glycol side chains for preventing nonspecific binding. (B) Photograph of the in situ electropolymerization setup, showing the three-electrode configuration within the PDMS well. (C) SEM image of the nanotube array formed after 60 s of electropolymerization at 1.2 V. (D) SEM image of the microstructured network evolved after 120 s of electropolymerization. (E) Transfer characteristics (*I*_d_–*V*_g_) measured at *V*_d_ = −0.5 V. (F) Corresponding transconductance (*g*_m_) profiles as a function of gate voltage.

This approach builds upon our previously established template-free electropolymerization methodology,^23^ which enables the formation of uniform and structurally tunable PEDOT-based nanotubes. Guided by this method, we first employed chronoamperometry to systematically evaluate the influence of applied potential and polymerization time on nanotube formation (Figure S2). Prior comparisons^23^ indicated that an applied potential of 1.2 V offers the most stable growth conditions, which effectively generates well-defined tubular structures, while avoiding the sparse morphologies typically formed at 1.1 V or the ruptured structures observed at 1.4 V due to excessively rapid polymerization. Therefore, 1.2 V was selected as the optimized electropolymerization potential in this work, with polymerization times of 60 and 120 s used to modulate structure size.

After assembling the PDMS well, electropolymerization was conducted using a three-electrode configuration, where the ITO source/drain pair served as the working electrode, an Ag/AgCl electrode acted as the reference, and a platinum wire functioned as the counter electrode (Figure 2Β). When electropolymerization was carried out at 1.2 V for 60 s, SEM images revealed the formation of highly ordered nanotube arrays (pore diameter = ∼876 nm, wall thickness = ∼275 nm, Figure S2G), characterized by tightly packed structures with uniform pore distribution (Figure 2C). These nanotubes exhibited a high surface area ratio, pronounced porosity, and vertically aligned channels, which facilitate efficient ion penetration, a critical factor for OECT operation in which PEDOT-based charge transport relies on the coordinated movement of injected ions and electronic carriers.

Extending the polymerization time to 120 s induced continued growth of the tube-like structures, which gradually expanded into thicker, intertwined microtubular or fibrous networks with increased wall thickness and overall structural volume. Compared to the nanotube group, this microstructured film possessed a substantially larger conductive polymer volume and longer ion/electron pathways, translating directly into enhanced volumetric capacitance and more efficient ion–electron coupling during device operation (Figure 2D).

The distinction between the nano- and microstructured channels extends beyond geometric differences and encompasses key material parameters such as channel-layer thickness, porosity and structural continuity, distribution of effective conductive pathways, and total surface availability for chemical functionalization. Together, these factors critically influence OECT transconductance, volumetric capacitance, and biosignal amplification, highlighting the significance of morphology engineering for high-performance sensing.

The structural distinction between nano- and microengineered channels profoundly impacts the device’s electrical performance, specifically the transconductance (*g*_m_), which quantifies the efficiency of ion-to-electron transduction. As shown in Figure 2E, the output transfer characteristics curves (*I*_d_–*V*_g_) exhibits a clear hierarchy: microstructure > nanotube > control. The microstructured group displayed the highest drain current due to the formation of denser conductive pathways capable of accommodating greater ionic injection. This trend is mirrored in the transconductance (Figure 2F), where the control group (planar PEDOT:PSS) exhibited the lowest *g*_m_, limited by its small active volume. Introducing the 60 s nanotube layer substantially increased *g*_m_ by expanding the porous conductive interface. However, the 120 s microstructured channel achieved the highest *g*_m_, attributed to its maximized volumetric capacitance resulting from the greater mass of electrodeposited polymer.

These results explicitly demonstrate that microstructural engineering is a decisive factor governing OECT performance. While the microstructured film (120 s) offers superior electrical amplification due to higher volumetric capacitance, the nanotube film (60 s) offers a unique balance of high surface area and open porosity, which may prove advantageous for minimizing steric hindrance during subsequent antibody functionalization. Thus, the ability to precisely tune this morphology provides a versatile handle for optimizing the sensitivity and dynamic range of the Nf-L biosensor.

### Verification of Antibody Immobilization and Mechanistic Analysis of Transconductance Suppression

To quantitatively validate the covalent immobilization of anti-Nf-L antibodies onto the channel surface, X-ray photoelectron spectroscopy (XPS) was employed to probe the surface chemical composition. We utilized the atomic ratio of nitrogen to sulfur (N/S) as a robust quantitative metric for antibody coverage. Since the protein backbone is rich in nitrogen (peptide bonds) but contains negligible sulfur (<1%), while the PEDOT copolymer is sulfur-rich, an increase in the N/S ratio serves as a direct signature of successful protein grafting.^31,32^

The XPS analysis revealed a striking morphological dependence on functionalization efficiency (Figure 3A). The nanotube array (60 s) exhibited the highest N/S ratio, indicating the most extensive antibody loading. This can be attributed to its unique topology: the open, vertically aligned nanotubular structure provides a significantly larger accessible surface area and porosity compared to the planar control, thereby maximizing the density of reactive carboxylic acid sites available for EDC/NHS coupling.

**Figure 3:**
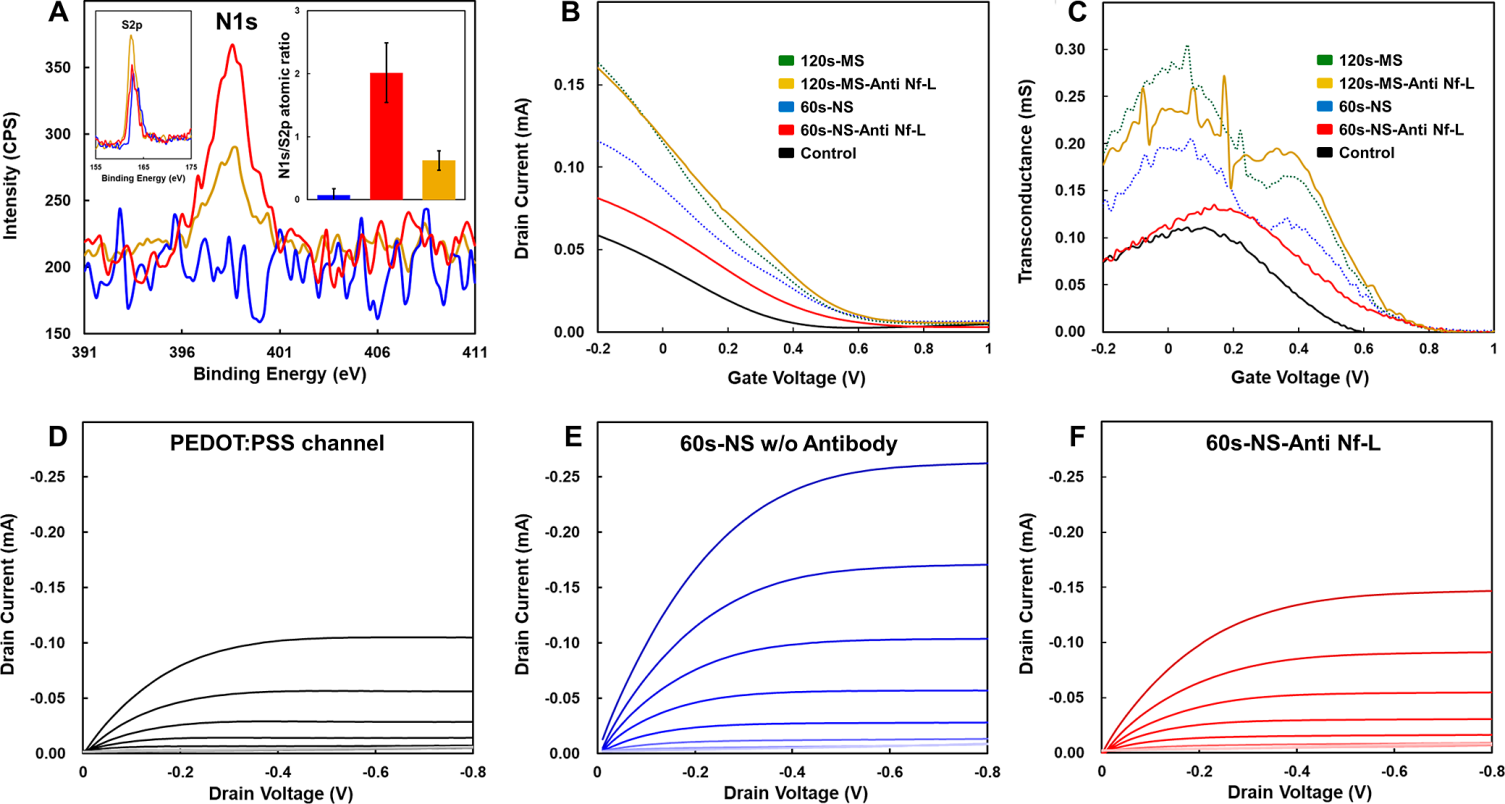
Spectroscopic validation and electrical characterization of antibody immobilization. (A) High-resolution XPS N 1s spectra and (inset) S 2p spectra. The bar chart (inset) compares the nitrogen-to-sulfur (N/S) atomic ratios. (B) Transfer characteristics (*I*_d_–*V*_g_) and (C) Transconductance (*g*_m_–*V*_g_) of the nanotube (blue/red) and microstructured (green/yellow) devices before (dotted lines) and after (solid lines) antibody functionalization. (D*–*F) Evolution of the output characteristics (*I*_d_–*V*_d_) throughout the fabrication process: (D) Pristine PEDOT:PSS control layer. (E) Nanotube-engineered channel after 60 s electropolymerization. (F) The same nanotube channel after anti-Nf-L immobilization, where the formation of an insulating antibody barrier reduces the drain current.

Conversely, while the microstructured film (120 s) possesses a larger total polymer volume, it displayed a substantially lower N/S ratio compared to the nanotube group. This suggests that as the nanotubes merge into dense fibrous networks, the effective surface-to-volume ratio decreases, or the intricate network induces steric hindrance that limits the diffusion of bulky antibody molecules into the deeper binding sites. The control group (nanotube without antibody) displayed negligible nitrogen signals, confirming that nonspecific physical adsorption is effectively minimized, likely due to the antifouling properties of the EG3 side chains.

The efficiency of antibody grafting was further corroborated by analyzing the modulation of the device’s electrical figures of merit. Upon antibody immobilization, the nanotube group exhibited the most pronounced suppression in both transconductance (Figure 3B,C) and drain current (Figure 3D–F). In mechanistic terms, the immobilized antibody layer acts as a dielectric barrier at the polymer/electrolyte interface. This insulating layer impedes the injection of ions from the electrolyte into the bulk PEDOT channel, thereby increasing the interfacial impedance and reducing the efficiency of ion-electron coupling (doping/dedoping process). Consequently, the magnitude of *g*_m_ reduction serves as an indirect measure of antibody density. The sharp decline observed in the nanotube device confirms the formation of a dense, insulating antibody layer. In contrast, the microstructured group experienced only a moderate reduction, consistent with its lower antibody density as indicated by XPS.

Taken together, the XPS, *g*_m_, and *I*_d_–*V*_d_ results collectively demonstrate that the correlation between chemical analysis and electrical suppression provides conclusive evidence that the EDC/NHS coupling chemistry enables efficient covalent grafting of antibodies onto the PEDOT copolymer channel. Notably, the 60 s nanotube architecture offers the optimal balance of surface accessibility and electrochemical performance. While the 120 s microstructure excels in intrinsic conductivity, the 60 s nanotube array provides superior biofunctionalization capacity. Therefore, the nanotube-engineered OECT was selected as the primary platform for the subsequent high-sensitivity detection of Nf-L. Furthermore, the output characteristics (*I*_d_–*V*_d_) confirmed that the device reaches the saturation regime at *V*_d_ = −0.5 V, consistent with prior studies.^23,25^ Consequently, this voltage was applied as the standard operating condition for the sensing measurements.

### Nf-L Sensing Performance: Concentration-Dependent Response and Ultrasensitive Detection

To ensure reliable biosensing, phosphate-buffered saline (PBS, 1×, pH 7.4) was selected as the supporting electrolyte. This physiological condition was strictly maintained to optimize the physicochemical state of all interface components: (1) Nf-L Stability: with an isoelectric point (pI) of ∼4.7,^33^ Nf-L retains a net negative charge at pH 7.4, which is essential for electrostatic interaction; (2) antibody bioactivity: the physiological pH preserves the native protonation state of antibody residues, ensuring maximum binding affinity;^9^ and (3) PEDOT redox stability: the neutral pH prevents the alkaline-induced dedoping of the conductive polymer, while the physiological ionic strength (∼150 mM) supports efficient volumetric ion gating.^34^

Prior to real-time sensing, we elucidated the signal transduction mechanism governing the Nf-L detection. To quantitatively validate how the immunocomplex formation influences the channel properties, we analyzed the concentration-dependent transconductance (*g*_m_) profiles (Figure S3A). The data reveals a systematic suppression of peak *g*_m_ with increasing antigen binding. The specific binding of Nf-L introduces a dense protein layer that acts as a dielectric barrier, effectively increasing the thickness of the electric double layer (EDL) at the polymer–electrolyte interface. This leads to a substantial reduction in the interfacial capacitance (*C*_int_). According to the OECT working principle (*g*_m_ ∝ μC*), this reduction in interfacial capacitance creates a series-impedance bottleneck that limits the effective volumetric capacitance (C*) available for gating.^35^ Consequently, the quantitative suppression of *g*_m_ verifies that the sensing signal arises from the modulation of ion injection efficiency via the capacitive blocking effect.

To evaluate the analytical performance of the engineered platform, we monitored the real-time amperometric response of the OECT immunosensor to varying concentrations of Nf-L. The device, biased at *V*_g_ = 0 V and *V*_d_ = −0.5 V, was subjected to sequential injections of Nf-L solutions ranging from 1 fg/mL to 1 ng/mL (in 1× PBS, pH 7.4). As illustrated in Figure 4A–C, the signal attenuation arises from the specific binding of Nf-L to the antibody-functionalized PEDOT channel. The formation of the antigen–antibody immunocomplex creates a dielectric passivation layer at the polymer–electrolyte interface. This insulating barrier effectively hinders the injection of hydrated ions into the bulk of the PEDOT copolymer, thereby suppressing the electrochemical doping process and reducing the channel conductivity.^36,37^

**Figure 4:**
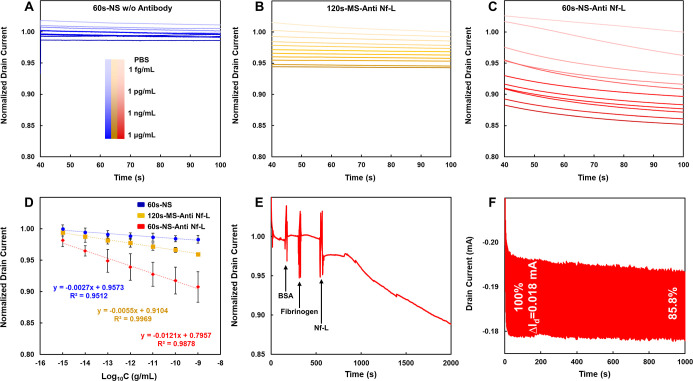
Sensing performance, calibration, and specificity. (A–C) Real-time normalized responses (*I*_d_/*I*_blank_) with applied *V*_g_ = 0 V (vs Ag/AgCl) and *V*_d_ = −0.5 V for (A) 60 s nanostructured, (B) 120 s microstructured (with antibody), and (C) 60 s nanostructured (with antibody) devices upon Nf-L addition. (D) Semilogarithmic calibration curves. The error bars represent the standard deviation (SD) of three independent measurements (*n* = 3). (E) Selectivity assay with *V*_g_ = 0 V (vs Ag/AgCl) and *V*_d_ = −0.5 V against BSA and Fibrinogen. (F) Operational stability over 500 cycles. The drain current response was recorded under pulsed gate voltage (*V*_g_ = 0.1 V (vs Ag/AgCl), 1 s pulse interval, *V*_d_ = −0.5 V).

A critical comparison of the two channel morphologies reveals a distinct hierarchy in sensing efficacy. While all antibody-modified devices displayed a monotonic current response, the 60 s nanotube architecture exhibited the most pronounced signal suppression (sensitivity). Statistical analysis of the normalized response (*I*_d_ */I*_blank_) confirmed that the nanotube group consistently outperformed the microstructured and control groups across the entire concentration range. This finding highlights a pivotal design principle: Sensing performance is not solely dictated by intrinsic transconductance. Although the 120 s microstructured group possessed a higher initial transconductance, as established in the previous section, its sensing response was inferior due to lower antibody loading density. Conversely, the nanotube group, with its superior surface-to-volume ratio and optimized pore accessibility, facilitated a higher density of immunocomplex formation, resulting in a stronger ion-blocking effect. The control device, lacking specific anchoring sites, showed a negligible signal change, confirming the selectivity of the response.

To assess the reproducibility of the sensing platform, three independently fabricated devices (*n* = 3) were tested across the full concentration range. The data is presented as the mean ± standard deviation (SD), confirming the acceptable batch-to-batch consistency and robustness of the OECT immunosensor. The corresponding calibration curves (Figure 4D) exhibit excellent linearity over a broad dynamic range of 1 fg/mL to 1 ng/mL, with correlation coefficients (*R* ^2^) of 0.9969 for the microstructured films and 0.9878 for the nanostructured film. The slightly lower *R* ^2^ in the nanostructured group is attributed to the stochastic nature of the template-free polymerization, which introduces minor morphological heterogeneity compared to the more uniform, coalesced network of the microstructured films. However, this trade-off is negligible given the substantial enhancement in sensitivity. The steeper slope of the anti-Nf-L conjugated nanotube group (Red) compared to the microstructured (Yellow) and nanotube without antibody group (Blue) groups quantitatively confirms its superior sensitivity. The amperometric response follows the linear regression equations

$$I_{d}{/I}_{blank}={-0.0554Log}_{10}\left[C_{Nf-L}\right]+0.9104\left(120s-MS-Anti-Nf-L\right)$$

$$I_{d}{/I}_{blank}={-0.1211Log}_{10}\left[C_{Nf-L}\right]+0.7957\left(60s-NS-Anti-Nf-L\right)$$

where *I*_d_ represents the measured drain current equilibrated after 100 s and *I*_blank_ represents the stabilized baseline current in PBS before analyte addition. The nanostructured device exhibited a sensitivity slope approximately 2.5-fold higher than that of the microstructured device. The microstructured film, characterized by a dense and continuous network, serves as a proxy for conventional planar PEDOT derivative films, similar to the planar control architecture established in our previous work.^23^ This comparison isolates the morphological influence, confirming that the performance enhancement is driven by the high surface-to-volume ratio of the template-free nanotubes rather than chemical composition. The LOD was determined using two distinct statistical approaches to ensure rigorous reporting. First, using the standard IUPAC 3σ criterion, the antibody-conjugated microstructured devices yielded an LOD of 0.70 fg/mL, whereas the nanostructured counterparts achieved a superior theoretical LOD of 0.062 fg/mL. It should be noted that this value is obtained via linear extrapolation from the lowest experimental concentration (1 fg/mL). While this indicates exceptional intrinsic sensitivity, the reliability of detection in the subfemtogram regime is theoretically projected.

To address the reliability of this extrapolation and explicitly account for device-to-device variation, we further applied the Hubaux-Vos method (ISO 11843-2).^38,39^ This rigorous statistical model, which considers the confidence intervals of the regression, yielded a conservative LOD of 32.77 fg/mL for the nanostructured device, compared to 41.47 fg/mL for the microstructured device (detailed formulas and parameters are provided in the Supporting Information). This confirms that even under strict statistical constraints, the sensor reliably detects Nf-L at levels significantly below the clinical cutoff in blood (∼10 pg/mL pg/mL).^40,41^

A critical analysis of the statistical performance reveals an engineering trade-off governed by the channel morphology. The microstructured devices, formed through a longer polymerization time, exhibit a more uniform film structure, resulting in lower device-to-device variation. In contrast, the template-free synthesis of the nanotube architecture introduces a higher degree of stochastic variation due to the random nature of nanotube growth. To quantify this trade-off, we scrutinized the effective detection resolution. Based on intrinsic noise (3σ/Slope), the nanotube device shows a superior theoretical resolution factor of 2.73 compared to 3.8 for the microstructured device. While the regression-based parameter (*S*_*y*/*x*_/Slope) currently yield a higher value of 40.27, compared to 12.98 for the microstructured device, this elevated dispersion is attributable to early stage fabrication variability rather than intrinsic sensor limitations. We anticipate that by refining the fabrication process and increasing the sample size, the standard error of the estimate (*S*_*y*/*x*_) will be reduced. Consequently, the practical resolution is expected to converge toward the low theoretical limit suggested by the intrinsic noise analysis.

Despite this trade-off in resolution, the Nanotube architecture remains the superior platform for ALS screening. In clinical diagnostics, the critical challenge is to reliably differentiate ALS patients (∼100 pg/mL) from healthy individuals. The Nanotube architecture’s massive signal amplification (sensitivity slope) effectively compensates for the device-to-device variation, securing a lower rigorous LOD (32.77 fg/mL). This detection limit is orders of magnitude lower than the healthy baseline (∼10 pg/mL), ensuring the sensitivity required to monitor the transition from physiological to pathological concentrations with high confidence.

To benchmark the performance of our Nanotube-engineered OECT against the current state-of-the-art, we compared our sensor with commercial ELISA-based method^19^ and recently reported transistor-based Nf-L sensors^9,30,42,43^ (summarized in Table S3). Our OECT architecture leverages volumetric capacitance, where the signal is amplified by ions penetrating the entire polymer bulk. Consequently, our platform achieves a theoretical LOD (0.062 fg/mL, ∼0.9 aM) and even a rigorous statistical LOD that are lower than existing transistor-based counterparts and comparable to commercial digital immunoassays (e.g., SiMoA).

In summary, the nanotube-engineered channel layer delivers the optimal balance of parameters for Nf-L sensing with the lowest LOD. These findings reaffirm that channel surface morphology, specifically the accessible nanotopography for biorecognition, plays a decisive role in biological signal amplification, often outweighing the contribution of bulk transconductance alone.

### Selectivity Assessment: Resistance to Nonspecific Interference and Evaluation of Device Stability and Repeatability

To validate the analytical specificity of the OECT immunosensor in complex biological environments, we conducted rigorous selectivity assays using high-abundance interfering proteins. As shown in Figure 4E, the sequential introduction of nontarget proteins, including bovine serum albumin (BSA) and Fibrinogen, elicited negligible signal drift, with the drain current remaining quiescent at the background level. These specific interfering agents were selected based on their dominance in blood physiology. BSA was chosen as it represents the most abundant protein, albumin, in plasma (∼35–50 mg/mL), constituting nearly 60% of the total protein content and serving as the primary source of background noise. Fibrinogen was selected as a rigorous model for biofouling due to its large molecular size and strong tendency to adsorb onto sensor surfaces.^38,44^ In sharp contrast, the introduction of Nf-L under identical operating conditions induced a rapid decrease in current within a few seconds, followed by a continuous signal decay as antigen–antibody binding progressed. These observations confirm that the device exhibits excellent specificity toward Nf-L, with rapid response kinetics capable of immediately reflecting the electrical perturbations caused by binding events at the channel surface.

This exceptional specificity is derived from the rational design of the dual-functional channel interface. First, the covalent grafting of anti-Nf-L antibodies via the EDOT-COOH moieties ensures high-fidelity molecular recognition, enabling precise discrimination between the target biomarker and interfering species. Second, and equally critical, the EDOT-EG3 side chains create a hydration layer that effectively suppresses hydrophobic interactions and minimizes the nonspecific physical adsorption of abundant serum proteins. The negligible response to these high-concentration challenges confirms that the hydration layer formed by the EDOT-EG3 moieties effectively suppresses biofouling interactions. Consequently, the nanotube-engineered OECT maintains high signal-to-noise ratios even in protein-rich environments, supporting its viability for analysis in complex clinical matrices such as plasma and serum.

Beyond sensitivity and selectivity, the reliability of the sensor readout is paramount. We evaluated the intra-assay repeatability by performing five consecutive measurements on a single device incubated with 1 ng/mL Nf-L. As shown in Figure S3B, the real-time current response curves overlap almost perfectly across all five cycles. A magnified view of the transient region further confirms consistent gating kinetics (Figure S3C). The calculated relative standard deviation (RSD) of the steady-state current was less than 0.1%, indicating negligible signal drift and high measurement precision. Furthermore, the operational stability of the nanotube channel was verified through rigorous cycle-to-cycle testing. The device retained stable current modulation over 500 continuous switching cycles (Figure 4F), confirming that the template-free electropolymerized nanotubes possess sufficient structural integrity to withstand repeated ionic doping/dedoping processes without mechanical degradation.

This sustained performance indicates that the nanotube architecture possesses excellent structural integrity, resisting mechanical collapse or delamination during prolonged electrochemical cycling. Furthermore, it confirms the stability of the covalent antibody tethering and the intrinsic robustness of the PEDOT copolymer backbone. Taken together, these results demonstrate that the nanotube-based OECT platform effectively rejects nonspecific interference while sustaining rigorous operational reliability, positioning it as a robust candidate for the longitudinal monitoring of neurodegenerative diseases.

Integrating the findings from fabrication, morphological characterization, electrical analysis, antibody immobilization, and Nf-L sensing performance clearly demonstrates that the nanostructured OECT outperforms both the microstructured and control devices across all key functional metrics. The nanotube architecture provides the highest surface area, greatest antibody loading density, and strongest biological signal amplification, collectively leading to superior Nf-L sensitivity, specificity, and operational stability. These results establish that the combination of nanostructured PEDOT copolymers with OECT-based transduction represents a highly promising platform for Nf-L biomarker detection, with significant potential for early diagnosis and disease monitoring in neurodegenerative disorders, as well as for further extension toward integrated bioelectronic system for advanced therapy and real-time physiological monitoring.^45^

## Conclusions

In this study, we successfully developed a high-performance OECT biosensor for the ultrasensitive detection of Nf-L by engineering a template-free PEDOT nanotube channel. The direct electropolymerization of poly­(EDOT–COOH–*co*-EDOT-EG3) created a hierarchical architecture that merges the benefits of high volumetric capacitance with a massive effective surface area for antibody immobilization.

In mechanistic terms, we confirmed via transconductance analysis that the sensing signal arises from the modulation of ion injection efficiency, where the immunocomplex formation acts as a dielectric barrier to perturb the interfacial capacitance. Statistically, we established the device performance using two approaches. While the nanostructured architecture achieves a theoretical intrinsic LOD of 0.062 fg/mL (IUPAC 3σ criterion), we further validated a rigorous LOD of 32.77 fg/mL using the Hubaux-Vos method to rigorously account for device-to-device variations.

Although the stochastic nature of the nanotube growth introduces a trade-off between sensitivity and effective resolution, the massive signal amplification provided by the nanotube architecture proves decisive for clinical utility. The achieved rigorous detection limit is orders of magnitude lower than the clinical baseline in healthy individuals (∼10 pg/mL), providing ample dynamic range to distinguish ALS-associated elevations from physiological levels. This work positions the nanotube-engineered OECT as a potent, label-free diagnostic platform capable of meeting the stringent demands of early neurodegenerative disease screening, with future potential for broader proteomic analysis.

Looking forward, we aim to further refine the precision of this platform by exploring a broader library of nanostructural morphologies to minimize measurement variance and enhance batch-to-batch consistency. Additionally, future studies will focus on validating the sensor’s performance in more complex clinical matrices, such as artificial blood and patient-derived plasma. Ultimately, we envision this nanotube-engineered OECT serving as a versatile, low-cost point-of-care diagnostic tool, facilitating early intervention and personalized monitoring for a spectrum of neurodegenerative disorders.

## Supplementary Material



## Notes

Experimental details regarding reagents, solvents, and instrumentation; synthetic schemes for the functionalized EDOT monomers; SEM images of the PEDOT-modified channel layers at various polymerization times; magnified views of the transient response during the initial and final stages of the stability test; equations and graphical determinations for the limit of detection (using both IUPAC 3σ and Hubaux-Vos methods); along with a table benchmarking the sensor’s performance against state-of-the-art platforms (PDF)

## Abbreviations

AD: Alzheimer’s disease
ALS: amyotrophic lateral sclerosis
BSA: bovine serum albumin
DCM: dichloromethane
DMSO: dimethyl sulfoxide
EDC: 1-ethyl-3-(3-(dimethylamino)­propyl)­carbodiimide
EDOT: 3,4-ethylenedioxythiophene
EGOFET: electrolyte-gated organic field-effect transistor
FET: field-effect transistor
GOPS: (3-glycidoxypropyl)­trimethoxysilane
IPA: isopropyl alcohol
ITO: indium tin oxide
LOD: limit of detection
MS: multiple sclerosis
Nf-L: neurofilament light chain
OECT: organic electrochemical transistor
PBS: phosphate-buffered saline
PDMS: polydimethylsiloxane
PEDOT: poly­(3,4-ethylenedioxythiophene)
POC: point-of-care
SEM: scanning electron microscopy
Sulfo-NHS: *N*-hydroxysulfosuccinimide
TBAP: tetrabutylammonium perchlorate
THF: tetrahydrofuran
