# Supplementary material for: Precision Channel Engineering of Nanotube-Embedded Organic Electrochemical Transistors for Ultrasensitive Neurofilament Light Chain Detection
Source: ACS Appl Bio Mater. 2026 Jan 23;9(4):2271–81. doi: 10.1021/acsabm.5c02404 (PMC12914629; doi:10.1021/acsabm.5c02404)
Supplement: Supplementary file 1 [file mt5c02404_si_001.pdf]

Supporting Information

Precision Channel Engineering of Nanotube-Embedded Organic Electrochemical Transistors for Ultrasensitive Neurofilament Light Chain Detection

Jia-Wei She[a,b,c], Lu-An Lin[d], Jayakrishnan Aerathupalathu Janardhanan[a], I-Chen Wang[a], Feng-Chen Hsu[e], Hsueh-Sheng Tseng[e], Yu-Sheng Hsiao[e], Hsiao-hua Yu*[a]*

[a] Smart Organic Materials Laboratory, Institute of Chemistry, Academia Sinica, No. 128, Section 2, Academia Road, Nankang, Taipei 11529, Taiwan

[b] Taiwan International Graduate Program (TIGP), Nano Science & Technology Program, Academia Sinica, No. 128, Section 2, Academia Road, Nankang, Taipei 11529, Taiwan

[c] Department of Engineering and System Science, National Tsing Hua University, No. 101, Section 2, Guangfu Road, East District, Hsinchu City, 300, Taiwan

[d] College of Medicine, Chang Gung University, No.259, Wenhua 1st Rd., Guishan Dist., Taoyuan City 33302, Taiwan

[e] Department of Materials Science and Engineering, National Taiwan University of Science and Technology, No. 43, Sec. 4, Keelung Rd., Taipei 106335, Taiwan

E-mail: yshsiao@mail.ntust.edu.tw; bruceyu@as.edu.tw

Experimental detail

Reagents and Solvents: EDOT-OH (95%+) was purchased from Angene Chemicals. Sodium hydride (60% dispersion in mineral oil), sodium hydroxide (98%), Amberlite® IR-120 (hydrogen form), 1-ethyl-3-(3-dimethylaminopropyl)carbodiimide hydrochloride (EDC·HCl, 99%), (3-glycidoxypropyl)trimethoxysilane (GOPS, 98%+), and deuterated solvents were obtained from Sigma–Aldrich. Methyl bromoacetate (98%+), sodium iodide (99%+), triethylene glycol (99%), pyridine (99%), methanesulfonyl chloride (98%), tetrabutylammonium perchlorate (TBAP, 99%+), and lithium perchlorate (99%) were acquired from Alfa Aesar. Trityl chloride (98%) and N-hydroxysulfosuccinimide sodium salt (Sulfo-NHS, 98%) were purchased from Acros Organics and Combi-Blocks, respectively. Common acids including hydrochloric acid (HCl, 37%) and sulfuric acid (95%+) were obtained from Fluka and Fisher Scientific. Aqueous PEDOT:PSS dispersion (Clevios PH1000) was sourced from Heraeus. Anhydrous solvents, such as N,N-dimethylformamide (DMF, 99.8%, Sigma–Aldrich), tetrahydrofuran (THF), and dimethyl sulfoxide (DMSO), were handled under a nitrogen atmosphere using standard inert gas techniques. All other solvents were of analytical grade and used as received.

Biological Reagents: Phosphate-buffered saline (PBS, 1×, pH 7.4) was obtained from Gibco (Thermo Fisher Scientific). The target Neurofilament Light chain antigen (Nf-L, cat# AG231), anti-Nf-L antibody (cat# MAB1615), fibrinogen (cat# 341573), and bovine serum albumin (BSA, cat# A9418) were all purchased from Sigma-Aldrich.

Characterization Instruments: Surface chemical composition was analyzed using a PHI-5000 VersaProbe X-ray photoelectron spectrometer (XPS). The morphologies of the electropolymerized films were investigated using a Zeiss Ultra Plus field-emission scanning electron microscope (FE-SEM) operated at an accelerating voltage of 10 kV. Surface roughness was evaluated using a Bruker AS-12VLR MM atomic force microscope (AFM). FTIR spectra were recorded using a PerkinElmer FT-IR spectrometer.

Substrate Preparation: Prior to device fabrication, Indium tin oxide (ITO)-coated glass substrates were cleaned via sequential ultrasonic agitation (30 min each) in detergent solution, acetone, dichloromethane (CH2Cl2), methanol (MeOH), isopropyl alcohol (IPA), and deionized water.

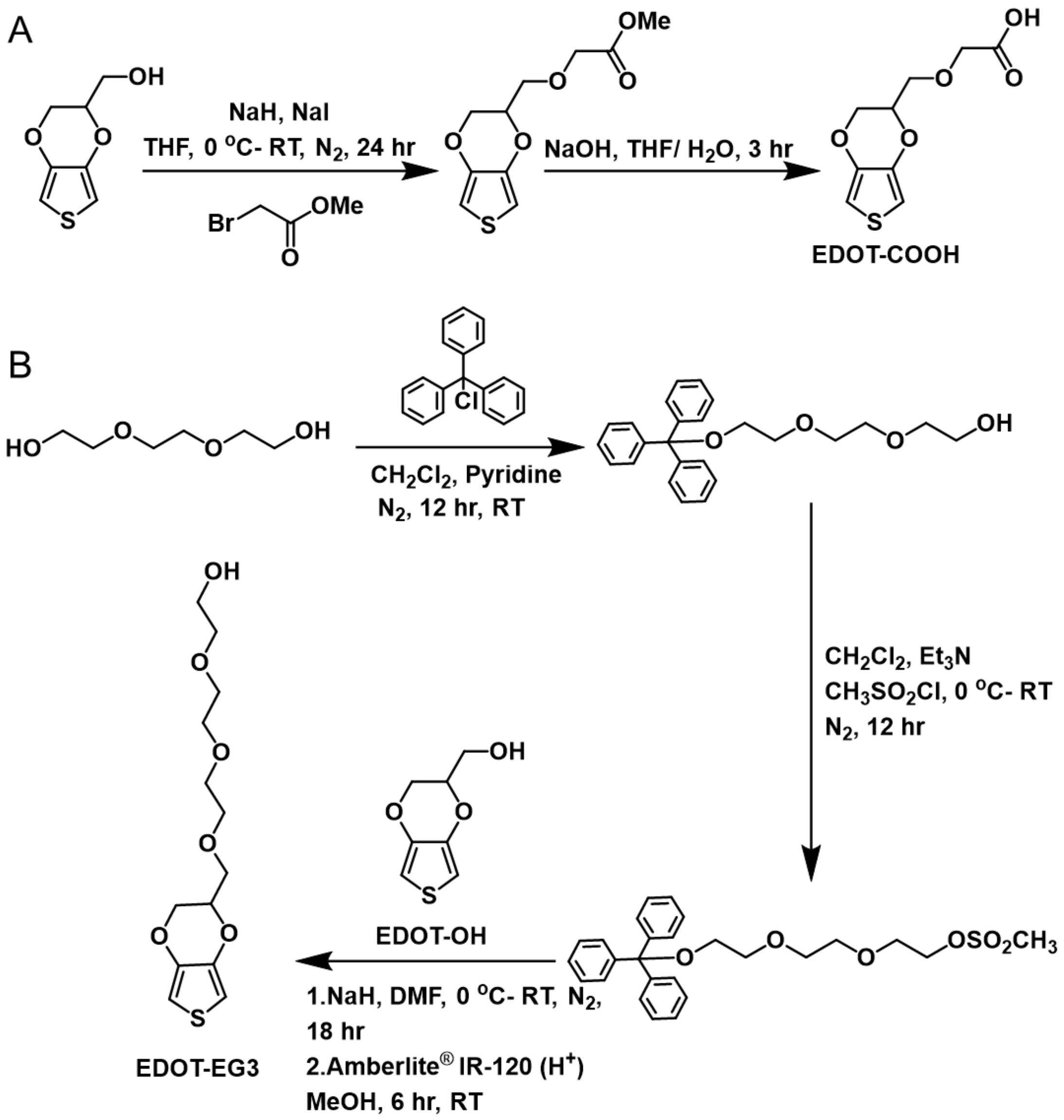

Figure S1. Synthetic schemes of functionalized EDOT monomers. (A) Synthesis of EDOT-COOH, designed for covalent antibody immobilization. The monomer was prepared via a two-step sequence involving the etherification of hydroxymethyl-EDOT (EDOT-OH) with methyl 2-bromoacetate, followed by base-catalyzed hydrolysis to liberate the reactive carboxylic acid moiety. (B) Synthesis of EDOT-EG3, designed to minimize non-specific protein adsorption. The precise multi-step pathway involves the initial mono-protection of triethylene glycol with a trityl group and subsequent mesylation to generate a reactive intermediate. This intermediate underwent nucleophilic substitution with EDOT-OH under basic conditions, followed by acidic deprotection using Amberlite IR-120 resin to yield the target hydroxyl-terminated EDOT-EG3.

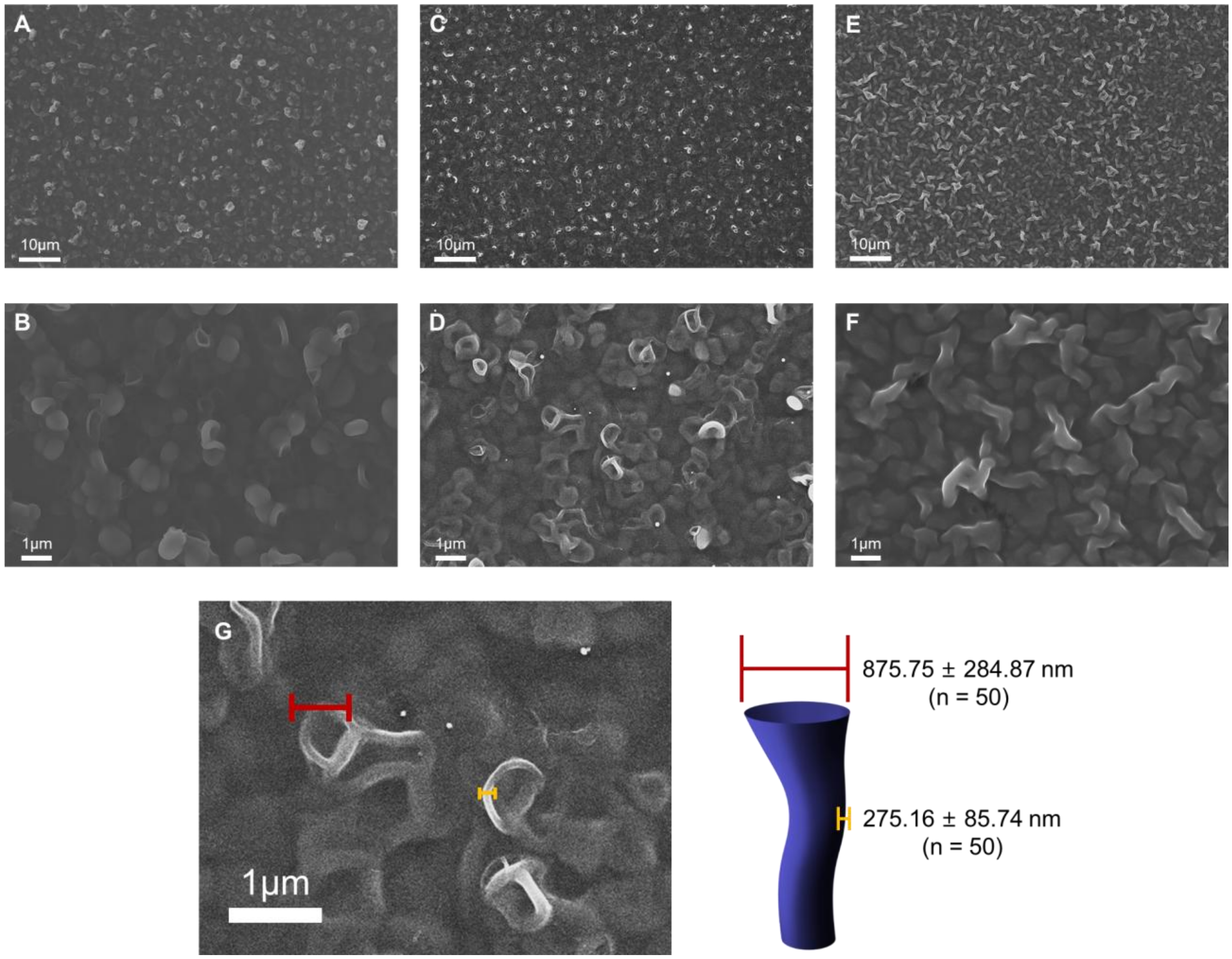

Figure S2. Time-dependent morphological evolution of the poly(EDOT-COOH-co-EDOT-EG3) channel interface. SEM micrographs showing the structural progression of copolymer films electropolymerized at 1.2 V (0-2°C) in dichloromethane for varying durations: (A, B) 30 s: The nascent stage of growth, characterized by a sparse distribution of nucleating structures with limited vertical extension and low areal density. (C, D) 60 s: The optimized nanotubular stage, exhibiting a uniform, high-density array of open-ended nanotubes. This morphology creates a highly porous topology with maximized surface-to-volume ratio, ideal for antibody loading. (E, F) 120 s: The microstructured stage, where prolonged polymerization leads to the coalescence of nanotubes into thicker, interconnected microtubular or fibrous networks, resulting in increased polymer volume but reduced porosity. (G) Quantitative characterization of the nanotubular morphology (n = 50), showing an average pore diameter of 875.75 ± 284.87 nm and a wall thickness of 275.16 ± 85.74 nm.

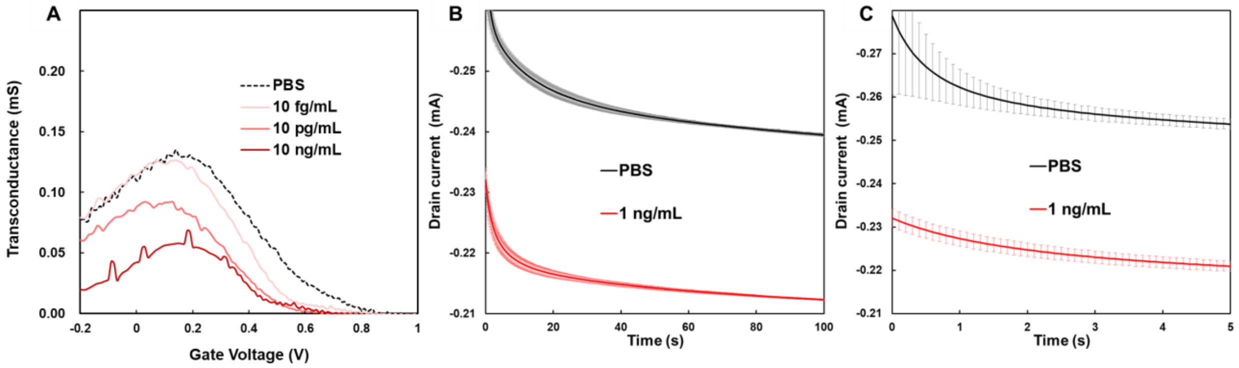

Figure S3. Mechanistic investigation and operational stability of the nanotube-embedded OECT. **(A)** Concentration-dependent transconductance (g_m) profiles of the antibody-functionalized 60s-NS device measured in PBS and varying concentrations of Nf-L. The systematic suppression of peak g_m with increasing analyte concentration indicates a reduction in the effective channel capacitance (C_{eff}) due to the formation of the insulating immunocomplex layer. **(B)** Intra-assay repeatability assessment showing five consecutive drain current (I_d) measurements (n = 5) performed on the same device in PBS (Black) and 1 ng/mL Nf-L (Red). **(C)** Magnified view of the transient response (0–5 s) from the same data. Despite initial transient fluctuations, the drain current trajectories exhibit excellent overlap, particularly reaching a highly stable steady state at t = 100 s with negligible, signal drift relative standard deviation (RSD)<0.1%, confirming the rigorous operational stability of the sensor for reliable quantification.

Determination of Limit of Detection (LOD) by IUPAC 3σ criterion

The Limit of Detection (LOD) was determined based on the standard deviation of the background response and the linear regression parameters derived from the calibration curve. According to the IUPAC 3σ criterion, the signal at the detection limit (y_{LOD}) is defined as the blank signal minus three times its standard deviation (for a signal decrease). By substituting this value into the linear regression equation ($y = S \cdot x + B$), the LOD was calculated using **the following equations**:

$$\log[\mathrm{LOD}] = \frac{(I_{\mathrm{blank}} - 3\sigma_{\mathrm{blank}}) - B}{S}$$

$$\mathrm{LOD} = 10^{\left(\frac{(I_{\mathrm{blank}} - 3\sigma_{\mathrm{blank}}) - B}{S}\right)}$$

Where I_{blank} represents the average normalized drain current of the blank sample (PBS). σ_{blank} is the standard deviation of the blank response ($n = 10$). S and B are the slope and intercept, respectively, determined from the linear regression of the calibration curve (ΔI_d vs. $\log C$).

Table S1. Analytical parameters and calculated LODs for the OECT immunosensors.

**Device Architecture**	I_{blank}	σ_{blank}	**S**	**B**	**log[LOD]**	**LOD (g/mL)**
**60s-NS**	0.99668	0.00224	−0.00269	0.9573	−12.119	7.597×10^{-12}
**120s-MS-anti Nf-L**	0.99749	0.00107	−0.00554	0.9104	−15.153	7.032×10^{-16}
**60s-NS-anti Nf-L**	0.99713	0.00176	−0.01211	0.7957	−16.206	6.226×10^{-17}

Determination of Limit of Detection (LOD) by Hubaux-Vos Method

The Limit of Detection (LOD) was calculated using the Hubaux-Vos method, a robust statistical approach that considers both the variance of the blank and the prediction interval of the calibration curve. The critical signal level ($I_{Critical}$) and the upper prediction band (Y_{upper}) were determined using the following equations:

$$Y_{upper} = (S \times X + B) + t_{exp} \times S_{y/x} \times \sqrt{1 + \frac{1}{N_{exp}} + \frac{(X - \bar{x})^2}{S_{xx}}}$$

$$I_{Critical} = I_{blank} - (t_{blank} \times \sigma_{blank})$$

Where LOD is defined as the concentration (X) at which the upper prediction band intersects with the critical signal level ($Y_{upper} = I_{Critical}$):

$$I_{Critical} = Y_{LOD} = (S \times log[LOD] + B) + t_{exp} \times S_{y/x} \times \sqrt{1 + \frac{1}{N_{exp}} + \frac{(log[LOD] - \bar{x})^2}{S_{xx}}}$$

The parameters used for the calculation were:

$N_{blank} = 10$, $t_{blank} = 1.8596$ (for 95% confidence), $\bar{x} = -10.5$, $S_{xx} = 247.5$, $t_{exp} = 1.729$

Table S2. Analytical parameters and calculated LODs for the OECT immunosensors.

Device Architecture	$I_{Critical}$	N_{exp}	t_{exp}	$S_{y/x}$	log[LOD]	LOD (g/mL)
**60s-NS**	0.9925	21	1.792	0.00634	−9.024	9.469×10^{-10}
**120s-MS-anti Nf-L**	0.9955			0.00616	−13.382	4.147×10^{-14}
**60s-NS-anti Nf-L**	0.9939			0.01943	−13.484	3.277×10^{-14}

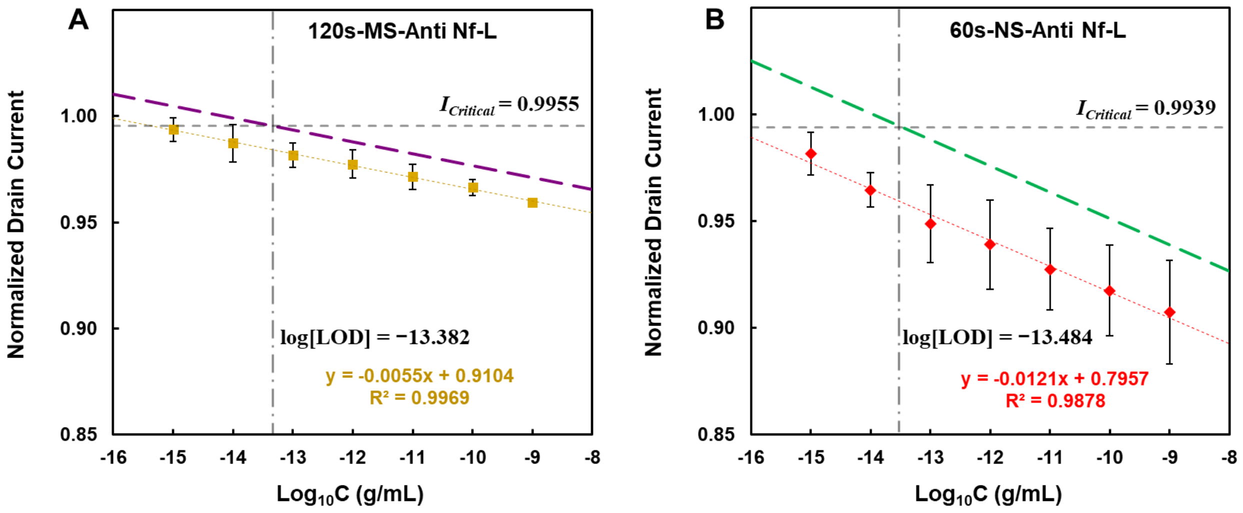

Figure S4. **Graphical determination of LOD using the Hubaux-Vos method.** The calibration curves for **(A)** 120s-MS-anti Nf-L and **(B)** 60s-NS-anti Nf-L devices. The dashed gray line represents the critical signal level ($I_{Critical}$), derived from the blank measurements. The dotted colored lines (purple for A, green for B) represent the upper prediction bands (Y_{upper}) of the regression models. The LOD is identified at the intersection of $I_{Critical}$ and the prediction band.

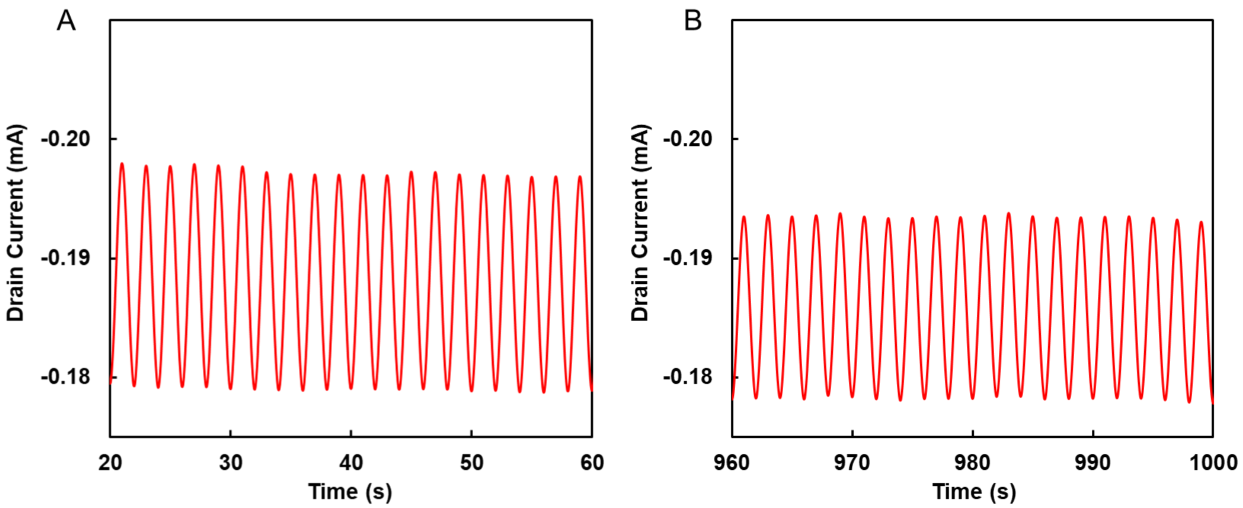

Figure S5. Operational stability characterization of the OECT device. Transient drain current response measured in PBS (1×, pH= 7.4) under pulsed gate modulation (V_g = 0.1 V (vs Ag/AgCl), 1 s pulse interval, V_d = −0.5 V). Magnified views showing the current oscillations during the (A) initial stage (20–60 s) and (B) final stage (960–1000 s) of the endurance test, confirming the preservation of device switching characteristics over prolonged operation.

Table S3. Comparison of analytical performance of the proposed OECT immunosensor with other Nf-L detection platforms.

Sensor Type / Method	Linear Range	Limit of Detection (LOD)	LOD Calculation Method	Reference
Single Molecule Array (SiMOA) / Ultrsensitive ELISA (Commercial Platform)	Not specified (Typically 4 logs)	55.2 fg/mL	Not specified (manufacturer data)	[19]
Electrolyte-Gated Organic Field-Effect Transistor (EGOFET) / Gate modified with anti-NfL antibody	100 fM – 10 nM	30 fM (~1.8 pg/mL)	3σ criterion	[9]
Extended-Gate Field-Effect Transistor (EGFET) / Vertical Si nanowires gate modified with NfL antibody	60 – 6×10^4 NDE/mL (NfL-specific Neuron-Derived Exosomes)	60 NDE/mL	Not stated	[30]
Graphene Field-Effect Transistor (G-FET) / Integrated microfluidics with Gate modified with anti-NfL antibody	100 fg/mL–1 ng/mL	55.63 fg/mL	3σ criterion	[42]
Graphene Field-Effect Transistor (gFET) / Channel modified with anti-NfL antibody	1 fg/mL – 10 pg/mL	0.66 fg/mL	3.3σ criterion	[43]
Organic Electrochemical Transistors (OECT) / Channel modified with PEDOT nanostructure and anti-NfL antibody	1 fg/mL – 10 ng/mL	0.062 fg/mL; 32.8 fg/mL	3σ criterion; Hubaux-Vos method	This work

Note: References correspond to the bibliography in the main manuscript.
